# Evaluation of Plaid Models in Biclustering of Gene Expression Data

**DOI:** 10.1155/2016/3059767

**Published:** 2016-03-09

**Authors:** Hamid Alavi Majd, Soodeh Shahsavari, Ahmad Reza Baghestani, Seyyed Mohammad Tabatabaei, Naghme Khadem Bashi, Mostafa Rezaei Tavirani, Mohsen Hamidpour

**Affiliations:** ^1^Biostatistics Department, Faculty of Paramedical Sciences, Shahid Beheshti University of Medical Sciences, Tehran 19716-53313, Iran; ^2^Medical Informatics Department, Faculty of Paramedical Sciences, Shahid Beheshti University of Medical Sciences, Tehran 19716-53313, Iran; ^3^English Language Department, Faculty of Paramedical Sciences, Shahid Beheshti University of Medical Sciences, Tehran 19716-53313, Iran; ^4^Proteomics Research Center, Faculty of Paramedical Sciences, Shahid Beheshti University of Medical Sciences, Tehran 19716-53313, Iran; ^5^Department of Hematology and Blood Banking, Faculty of Paramedical Sciences, Shahid Beheshti University of Medical Sciences, Tehran 19716-53313, Iran

## Abstract

*Background.* Biclustering algorithms for the analysis of high-dimensional gene expression data were proposed. Among them, the plaid model is arguably one of the most flexible biclustering models up to now.* Objective.* The main goal of this study is to provide an evaluation of plaid models. To that end, we will investigate this model on both simulation data and real gene expression datasets.* Methods.* Two simulated matrices with different degrees of overlap and noise are generated and then the intrinsic structure of these data is compared with biclusters result. Also, we have searched biologically significant discovered biclusters by GO analysis.* Results.* When there is no noise the algorithm almost discovered all of the biclusters but when there is moderate noise in the dataset, this algorithm cannot perform very well in finding overlapping biclusters and if noise is big, the result of biclustering is not reliable.* Conclusion.* The plaid model needs to be modified because when there is a moderate or big noise in the data, it cannot find good biclusters. This is a statistical model and is a quite flexible one. In summary, in order to reduce the errors, model can be manipulated and distribution of error can be changed.

## 1. Introduction

In biology, the cell is the basic structure of any organism. All cells of an organism have the same genes that could be at different expression levels across numerous conditions [1]. Scientists have concluded that different conditions could affect it in terms of whether a particular gene is expressed and how it could be expressed. The organism's health may be compromised due to the different expressions present. So it seems crucial to evaluate the levels of genome when exposed to tense factors [2]. In recent years, DNA microarray technology has provided monitoring of thousands of gene expressions simultaneously when cells are under different conditions and various processes. This technology has a key role in accelerating and increasing the efficiency of gene expression studies [3]. The development of this technique has led to the availability of gene expression matrix with rows containing thousands of genes and columns containing hundreds of conditions [4]. Clustering has been one of the most important techniques used for detecting pattern recognition and could find groups with similar expression patterns [3]. In gene expression data, some genes usually behave similarly under a subset of conditions and therefore these genes may not be expressed in other conditions. Furthermore, genes could be expressed in more than one subset. Therefore, traditional clustering methods will fail to discover such patterns [5]. In order to overcome these constraints and for the purpose of finding the appropriate gene expression patterns, biclustering methods have been proposed of which computational framework is more flexible [6]. A bicluster is a subset of genes that has similar expression patterns over a subset of conditions; so biclustering methods have determined homogeneous submatrices [7]. The first biclustering algorithm, the so-called block clustering, has been developed by [8]. Cheng and Church proposed the first biclustering algorithm for the analysis of high-dimensional gene expression data [9]. Since then, many different biclustering algorithms have been developed. Currently, there exists a diverse spectrum of biclustering tools that follow different algorithmic concepts basis on type of biclusters and definition of patterns [10]. Each of these algorithms has been proposed on the basis of coherence patterns and therefore based on these patterns, different submatrices have been identified. For instance, plaid model finds constant value biclusters, Cheng and Church (CC) model finds constant row biclusters, and OPSM and ISA find coherent evolutions biclusters [11]. And yet, there are some common issues with biclustering algorithms in general. Noise/errors in the data are the first issue that limits the discovery of appropriate biclusters [12]. The second issue would be the ability of algorithms to find overlapping biclusters [13]. Therefore, an important question is whether the algorithms based on these issues can find valid biclusters. Most of these algorithms have ignored noise and discovered biclusters based on all of gene expression data [14]. Also some of them could not find overlapping biclusters [13].

Distribution parameter identification is one of biclustering algorithms in which it is assumed that the data structures follow a statistical model and then trying to fit its parameters to the data by minimizing a certain criterion through an iterative approach is done [15]. Plaid models, spectral biclustering, and rich probabilistic model are some examples of this kind of biclustering. Among them, the plaid model is arguably one of the most flexible biclustering models up to now. This algorithm describes the biclustering structure of the data matrix. This model is proposed by [16, 17] and modified by [18]. It defines the expression levels as a sum of layers, constructed as biclusters. The main goal of this study is to provide a systematic evaluation of plaid models. To that end, we will investigate this model on both simulation data and real gene expression datasets by validity indices.

## 2. Methods

### 2.1. Overview of Plaid Model

The plaid model is a model based on biclustering approach that is used for analysis of gene expression data. This is a statistical model and assumes that the level of matrix entries is sum of the uniform backgrounds and *k* biclusters. So the expression matrix with *I* genes (rows) and *J* conditions (columns) is represented as

(1) $$\begin{array}{c} Y_{ij}=\mu_{0}+\overset{K}{\underset{k=1}{\sum }}\theta_{ijk}\rho_{ik}\kappa_{jk}, \end{array}$$

where *μ*_0_ is a general matrix background and *θ*_*ijk*_ = *μ*_*k*_ + *α*_*ik*_ + *β*_*jk*_ and *μ*_*k*_ is the added background in bicluster *k* and *α* and *β* are column specific additive constants in bicluster *k*. Also, *ρ*_*ik*_ ∈ {0,1} and *κ*_*jk*_ ∈ {0,1} are gene-bicluster membership and condition-bicluster membership indicator variables. The general biclustering problem is now formulated as finding parameters values so that the resulting matrix would fit the original data as much as possible. Formally, the problem is minimizing of ∑_*ij*_[*Y*_*ij*_ − ∑*θ*_*ijk*_ *ρ*_*ik*_ *κ*_*jk*_]^2^.

### 2.2. Validation of Model

#### 2.2.1. Simulation Data

Two simulating matrices with different degrees of overlap and noise are generated and then the intrinsic structure of these data is compared with biclusters result. We embedded two biclusters in the matrices with overlapping degrees of 0%, 10%, and 25% and noise degrees of 0%, 1%, 3%, 5%, and 10%. For the purpose of this study, simulations data were included in matrices with sizes 50*∗*20 and 500*∗*50 and distribution of *N*  (0,100) that embedded two biclusters as normal distribution with means of 3 and 9 and variance 0.1, respectively. So other entries are built with *N*  (0,100). Noise was built in the data with binary distribution and then values were generated by normal distribution with mean of 20 and variance of 4. We evaluated performance of the plaid algorithms based on three criteria that are numbers of rows and columns and overlap degree of biclusters. We ran the algorithm for 1000 iterations and the averages criteria were reported.

Table 1 lists statistics for rows and columns number of generated biclusters.

#### 2.2.2. Biological Significant

The result of the different biclustering techniques in microarray data is groups of genes, coexpressed with each other strongly, so we expect these genes to have the same functions. Gene ontology biological process could be the function that measures these similarities and covers three domains: cellular component, molecular function, and biological process. GO enrichment validation is a hypergeometric test for GO enrichment. This statistical test is significant if the genes in the biclusters are annotated with GO terms and are not specified by chance. So for the purpose of evaluating the quality of biclusters we have applied plaid algorithm to real dataset and searched biologically significant discovered biclusters in the Database for Annotation, Visualization and Integrated Discovery (DAVID) bioinformatics resources [19]. The real dataset is related to breast cancer (docetaxel resistance) article in 2005 that was included in CGED [20]. 44 breast tumor tissues were sampled through biopsy. Numbers of assayed genes were 2453.

## 3. Result

### 3.1. Simulation Data

In this section, we implemented the plaid algorithms on two simulate datasets and then evaluated them. In this study two R packages, Biclust and Bioconductor, were used. As shown in Table 1, this algorithm was applied to matrix with size of 50 × 20. When there was no noise, the algorithm was capable of discovering biclusters with different degrees of overlap and results exactly have corresponded to what was generated. It could be stated that plaid model is efficient in this case. But when there was a little noise, almost 0.01%, about 20–50% of biclusters could not be found correctly and also when there was an overlap among biclusters, these algorithms could not discover it. When noise was larger, plaid algorithm could not identify any biclusters.

**Figure 1:**
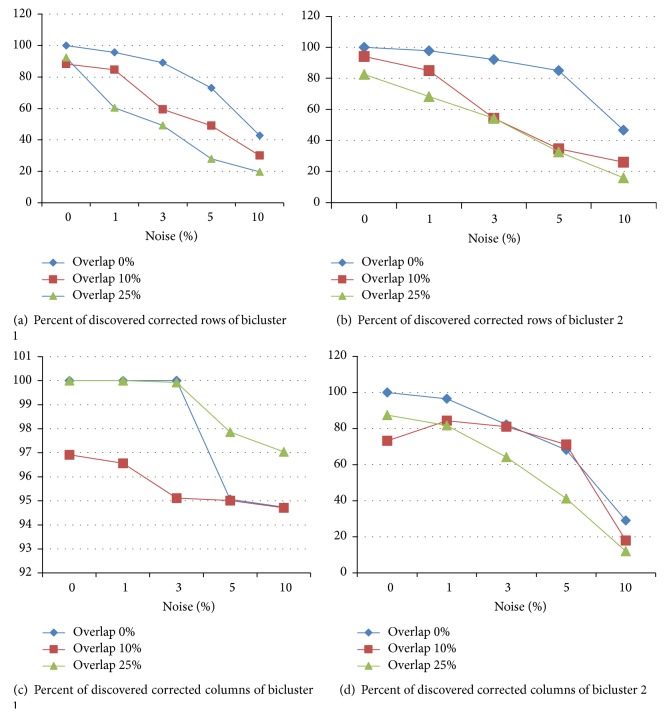
Corrected rows and columns of discovered biclusters in matrix with dim 500 × 50.

As shown in Table 2, for matrix with size of 500 × 50 when there was no noise and overlap in data, the algorithm could discover all biclusters correctly. When overlap degrees were 10% or 20%, algorithm performed well, and yet it could discover 90% of elements. Anytime there were 0.01%, 0.03%, and 0.05% noise in dataset, this algorithm could correctly discover 80%, 40–50%, and less than 40% of biclusters, respectively. When noise was 10%, this algorithm could not find the biclusters correctly and the most of the biclusters were ignored. Also when there was noise in the data, this algorithm could not discover the overlapped biclusters.  Figure 1 shows the percent of corrected rows and columns of biclusters in the matrix with dim 500 × 50. As shown here with the larger amount of noise, the diagnosis of the corrected biclusters reduced, especially when there is an overlap among biclusters.

### 3.2. Real Data

First, datasets are normalized with median approach and then missing values are computed with *k*nn (*k* Nearest Neighborhood) method. In our experiment we found 5 biclusters with the size of minimum 3 and maximum 189. Information about the discovered biclusters is shown in Table 3. In this table the first column contains the label of each bicluster. The second and third columns report the number of genes and conditions, respectively, and the last column contains the mean square residue of the biclusters.

Table 4 shows the significant GO terms for the set of genes that is discovered by each result of the biclusters along with their *P* value. We used the web tool DAVID to evaluate the discovered biclusters. For each bicluster, we first denoted numbers of GO term and then evaluated the significance of the functions.

## 4. Discussion

Many biclustering algorithms and models have been already proposed. Till now, one of the most flexible biclustering models is the plaid model [21].

In order to evaluate the plaid model in biclustering of gene expression data statistically, we generated two datasets with different noise and overlap and used a real dataset. Then these items were considered through statistical and biological criteria. Obviously, this algorithm can perform well when size of data is small and there is no noise. In this case, the algorithm is capable of discovering biclusters with different degrees of overlap. Nonetheless, when there is a little noise, this algorithm cannot discover all biclusters correctly and most of the information is ignored. Likewise, when noise is large, it cannot identify any biclusters. For matrix with size of 500 × 50, biclusters are well discovered when there is not any noise in data; consequently, it is almost capable of finding overlap biclusters. When there are little (0.01%) and moderate noise (0.03, 0.05%) in dataset, this algorithm cannot perform well in finding overlapping biclusters and if noise is big, result of biclustering is not reliable. For the purpose of biological evaluation, we used plaid model for a breast cancer dataset containing 2243 genes and 70 conditions. In this study we found 5 biclusters whose MSR measures are small, leading to their acceptance in the experiment. For each bicluster, we checked number of GO terms. Minimum and maximum numbers of GO terms are 10 and 748 which stand for biclusters with 3 and 189 genes. As a result, A, B, and D biclusters are highly enriched and the largest biclusters are more acceptable. Perhaps biclusters with very small genes could not be accounted for and should be rejected.

Most of the researches which used biclustering are concerned with the introduction of a new approach, while only a few of them have evaluated existing methods especially plaid model. Also, in most of these studies, the evaluations have been done on gene expression datasets, not on simulated data. Prelic et al. in 2006 evaluated 5 biclustering algorithms including CC [9], SAMBA [3], OPSM [22], ISA [23], and xMotif [24]. This study showed that ISA and SAMBA discovered 80% of biclusters correctly and CC and xMotif less than 40% on noise-free data, but while noise level increased more than 5%, the efficiency of all algorithms extremely decreased except ISA which is robust to noise [4]. Eren et al. in 2012 compared 12 biclustering algorithms by using synthetic data and demonstrated that no algorithm was able to fully separate biclusters with substantial overlap and also showed that algorithms which are model based seem more robust to noise than the others. At the end, they found the highest proportion of enriched biclusters in gene ontology analysis [25].

## 5. Conclusions

In this study, we evaluated the capabilities of plaid algorithm to identify biologically significant groups of coexpressed genes under a number of conditions. The evaluation criteria of biological significance for biclusters used in our study were GO annotation and simulation studies. GO enrichment analysis showed that biological significance of each bicluster is high, especially when the size of biclusters is big. The purpose behind this study was to evaluate plaid model based on different degrees of noise and overlap. Results show that when there is not any noise in the data, the algorithm can correctly discover biclusters with overlap. When there is a little noise in data and matrix of data is small algorithm could not find biclusters properly; yet if matrix is large it can recognize the biclusters properly. Furthermore, when there is a big noise in the data, algorithm could not discover biclusters and results are not reliable. Both the simulation studies and the real data analysis have demonstrated that the plaid algorithm is suitable for discovering patterns and provided useful information for researchers in big datasets with little noise.

There are some issues which should be considered while using plaid model. The plaid model is a statistical model and a quite flexible one, so it can be improved and used in genomic studies.

This model was constructed based on normal distribution and the parameters were estimated through minimizing the least squares criterion. But when the data is not normally distributed, the least squares criterion seems to be inefficient [26]. The distribution of the normalized gene expressions often has heavy tails and asymmetry. Traditional centering and scaling indexes in normal distribution approximations including the mean and the standard deviation are sensitive to outliers [27]. So normal distribution is not efficient when the data is noisy and it is better to use Laplace distribution in plaid model while it uses median as a location parameter and the scale parameter with the mean absolute deviation which is robust to noise and outliers.

Also, the results show that almost more than 80% of overlapped elements can be discovered in noise-free data by plaid model. But noises in data cause problems in discovery of overlap among biclusters. So considering Laplace distribution which causes the algorithm to be robust to noise it improves the algorithm for discovery of overlap among biclusters.

## Figures and Tables

**Table 1:** Mean and MSR of numbers of discovered rows and columns of biclusters in matrix 50 *∗* 20.

Scenario		Num of True Bic	Dimensions of True biclusters	Num of discovered biclusters (MSR)	Num of correct rows (MSR)		Num of correct columns (MSR)		Num of overlap discovered (MSR)
Noise %	Overlap				Bic1	Bic2	Bic1	Bic2	
	*N* (%)								
**0**	**0 (0)**		**21 × 6**,** 12 × 5**	2.31 (1.09)	21.0 (0.00)	12.0 (0.00)	6 (0.00)	5 (0.00)	0 (0.00)
**0**	**6 (10)**	**2**	**20 × 6**,** 12 × 5**	2.29 (0.84)	20.0 (0.00)	12.0 (0.00)	6 (0.00)	5 (0.00)	6 (0.00)
**0**	**10 (20)**		**20 × 6**,** 12 × 5**	2.45 (0.68)	20.0 (0.00)	22.0 (0.05)	6 (0.00)	5 (0.00)	10 (0.00)

**1**	**0 (0)**		**21 × 6**,** 12 × 5**	1.95 (2.27)	10.6 (52.08)	10.6 (8.09)	4.7 (2.16)	4.6 (1.08)	0 (0.00)
**1**	**6 (10)**	**2**	**20 × 6**,** 12 × 5**	2.00 (2.32)	11.5 (64.47)	10.6 (9.93)	4.4 (3.03)	4.6 (2.95)	0 (0.00)
**1**	**11 (20)**		**20 × 6**,** 12 × 5**	1.80 (1.38)	18.7 (128.19)	10.5 (25.78)	4.7 (2.38)	4.8 (4.21)	0 (0.00)

**Table 2:** Mean and MSR of numbers of discovered rows and columns of biclusters in matrix 500 *∗* 50.

Scenario		Num of bicluster	Dimensions of biclusters	Num of discovered biclusters (MSR)	Num of correct rows (MSR)		Num of correct columns (MSR)		Num of overlap discovered (MSR)
Noise %	Overlap				Bic1	Bic2	Bic1	Bic2	
	*N* (%)								
**0**	**0 (0)**		**101 × 20**,** 121 × 20**	2.82 (1.93)	101.00 (0.00)	121.00 (0.00)	20.00 (0.00)	19.44 (1.42)	0.00 (0.00)
**0**	**35 (10)**	**2**	**121 × 20**,** 205 × 20**	4.29 (9.59)	110.21 (54.15)	195.07 (74.43)	19.94 (1.20)	15.56 (8.73)	28.81 (6.19)
**0**	**76 (20)**		**196 × 20**,** 184 × 20**	4.32 (7.05)	185.26 (87.97)	151.79 (82.26)	20.00 (0.00)	18.37 (12.65)	37.16 (38.84)

**1**	**0 (0)**		**101 × 20**,** 121 × 20**	3.39 (3.05)	99.92 (2.53)	119.46 (0.00)	20.00 (0.00)	18.51 (10.63)	0.00 (0.00)
**1**	**35 (10)**	**2**	**121 × 20**,** 205 × 20**	6.03 (16.56)	101.91 (64.86)	179.22 (68.22)	19.94 (1.16)	18.29 (17.07)	20.38 (14.62)
**1**	**76 (20)**		**196 × 20**,** 184 × 20**	4.92 (11.15)	167.81 (98.41)	135.50 (83.37)	20.00 (0.00)	17.37 (22.19)	23.85 (52.15)

**3**	**0 (0)**	**2**	**101 × 20**,** 121 × 20**	4.73 (9.08)	92.91 (8.55)	112.19 (12.58)	20.00 (0.00)	16.24 (29.54)	0.113 (0.113)
**3**	**35 (10)**		**121 × 20**,** 205 × 20**	6.21 (21.02)	86.72 (140.16)	156.87 (156.38)	19.78 (4.14)	17.59 (16.75)	11.65 (23.35)
**3**	**76 (20)**		**196 × 20**,** 184 × 20**	5.22 (13.08)	136.57 (412.83)	127.25 (289.13)	19.99 (0.00)	14.74 (32.36)	15.64 (60.36)

**5**	**0 (0)**		**101 × 20**,** 121 × 20**	4.77 (9.21)	81.66 (420.92)	102.06 (586.21)	19.93 (0.07)	14.33 (85.61)	0.87 (0.86)
**5**	**35 (10)**	**2**	**121 × 20**,** 205 × 20**	6.05 (20.15)	75.95 (490.59)	140.39 (392.47)	19.67 (5.19)	15.66 (34.91)	8.75 (26.25)
**5**	**76 (20)**		**196 × 20**,** 184 × 20**	5.16 (12.88)	109.19 (956.23)	123.07 (883.16)	19.73 (0.03)	10.35 (59.34)	11.70 (64.29)

**10**	**0 (0)**		**101 × 20**,** 121 × 20**	4.16 (7.03)	48.92 (276.52)	94.98 (426.82)	19.52 (1.25)	8.96 (192.06)	4.22 (6.26)
**10**	**35 (10)**	**2**	**121 × 20**,** 205 × 20**	5.06 (12.67)	56.53 (434.02)	137.14 (513.55)	19.53 (4.49)	12.19 (151.78)	13.93 (21.07)
**10**	**76 (20)**		**196 × 20**,** 184 × 20**	4.38 (9.73)	73.65 (784.51)	90.06 (963.49)	19.52 (1.04)	8.92 (218.00)	3.08 (72.92)

**Table 3:** Information about bicluster result.

Label	Genes	Conditions	MSR
A	189	2	56.98
B	78	6	21.06
C	14	9	25.73
D	30	6	0.23
E	3	13	0.00

**Table 4:** Biological significant of biclusters result.

Bicluster	Number of GO terms	Ontology	*P* value			
			<0.05	<0.01	<0.005	<0.001
A	748	Biological process	87.5	63.1	57	42.5
		Molecular function	85.5	67.3	60	53.6
		Cellular component	84.2	58.4	47.5	28.7

B	94	Biological process	76.5	58.8	51	7.8
		Molecular function	81	38.1	38.1	23.8
		Cellular component	63.6	50	36.4	9.1

C	43	Biological process	57.7	19.2	15.4	11.5
		Molecular function	40	20	20	10
		Cellular component	71.4	14.3	14.3	14.3

D	171	Biological process	96	58	41.7	22.5
		Molecular function	88.9	72.2	61.1	50
		Cellular component	87.9	60.6	54.5	33.3

E	10	Biological process	80	0	0	0
		Molecular function	—	—	—	—
		Cellular component	60	20	0	0

## References

[1] Crick F. (1970). Central dogma of molecular biology. *Nature*.

[2] Jae K. L. (2001). Analysis issues for gene expression array data. *Clinical Chemistry*.

[3] Tanay A., Sharan R., Shamir R. (2002). Discovering statistically significant biclusters in gene expression data. *Bioinformatics*.

[4] Prelić A., Bleuler S., Zimmermann P. (2006). A systematic comparison and evaluation of biclustering methods for gene expression data. *Bioinformatics*.

[5] Yang M. S. (1993). A survey of fuzzy clustering. *Mathematical and Computer Modelling*.

[6] Gan X., Liew A. W.-C., Yan H. (2008). Discovering biclusters in gene expression data based on high-dimensional linear geometries. *BMC Bioinformatics*.

[7] Tanay A., Sharan R., Shamir R. (2004). Biclustering algorithms: a survey. *Science*.

[8] Hartigan J. A. (1972). Direct clustering of a data matrix. *Journal of the American Statistical Association*.

[9] Cheng Y., Church G. M. Biclustering of gene expression data.

[10] Pontes B., Giráldez R., Aguilar-Ruiz J. S. (2013). Configurable pattern-based evolutionary biclustering of gene expression data. *Algorithms for Molecular Biology*.

[11] Madeira S. C., Oliveira A. L. (2004). Biclustering algorithms for biological data analysis: a survey. *IEEE/ACM Transactions on Computational Biology and Bioinformatics*.

[12] Raser J. M., O'Shea E. K. (2005). Noise in gene expression: origins, consequences, and control. *Science*.

[13] Wang R., Shanghai T. U., Duqian M., Li G., Zhang H. Rough overlapping biclustering of gene expression data.

[14] Gupta R., Rao N., Kumar V. (2011). Discovery of error-tolerant biclusters from noisy gene expression data. *BMC Bioinformatics*.

[15] Hochreiter S., Bodenhofer U., Heusel M. (2010). FABIA: Factor Analysis for Bicluster Acquisition. *Bioinformatics*.

[16] Lazzeroni L., Owen A. (2002). Plaid models for gene expression data. *Statistica Sinica*.

[17] Liu X., Wang L. (2007). Computing the maximum similarity biclusters of gene expression data. *Bioinformatics*.

[18] Turner H., Bailey T., Krzanowski W. (2005). Improved biclustering of microarray data demonstrated through systematic performance tests. *Computational Statistics & Data Analysis*.

[19] Sherman B. T., Huang D. W., Tan Q. (2007). DAVID Knowledgebase: a gene-centered database integrating heterogeneous gene annotation resources to facilitate high-throughput gene functional analysis. *BMC Bioinformatics*.

[20] Kato K., Yamashita R., Matoba R. (2005). Cancer Gene Expression Database (CGED): a database for gene expression profiling with accompanying clinical information of human cancer tissues. *Nucleic Acids Research*.

[21] Eren K., Deveci M., Küçüktunç O., Çatalyürek Ü. V. (2013). A comparative analysis of biclustering algorithms for gene expression data. *Briefings in Bioinformatics*.

[22] Ben-Dor A., Chor B., Karp R., Yakhini Z. (2003). Discovering local structure in gene expression data: the order-preserving submatrix problem. *Journal of Computational Biology*.

[23] Bergmann S., Ihmels J., Barkai N. (2003). Iterative signature algorithm for the analysis of large-scale gene expression data. *Physical Review E—Statistical, Nonlinear, and Soft Matter Physics*.

[24] Murali T. M., Kasif S. Extracting conserved gene expression motifs from gene expression data.

[25] Caldas J., Kaski S. Bayesian biclustering with the plaid model.

[26] McCullagh P., Nelder J. A. (1989). *Generalized Linear Models*.

[27] Yang Y. H., Dudoit S., Luu P., Speed T. P., Bittner M. L., Chen Y., Dorsel A. N., ougherty E. R. Normalization for cDNA microarray data.

